# Biventricular hypertrabeculations or noncompaction in a 54‐year‐old woman in Sub‐Saharan Africa: A case report

**DOI:** 10.1002/ccr3.2658

**Published:** 2020-01-14

**Authors:** Ahmadou Musa Jingi, Liliane Mfeukeu‐Kuate, Sylvie Ndongo Amougou, Jerome Boombhi, Herbert Hakapoka, Pierre Mintom, Edvine Wawo‐Guela, Ba Hamadou

**Affiliations:** ^1^ Department of Internal Medicine and Specialties Faculty of Medicine and Biomedical sciences University of Yaounde 1 Yaounde Cameroon; ^2^ Centre Médical d'Hippodrome (CMH) Yaounde Cameroon; ^3^ Clinical Research, Education, Networking, and Consultancy (CRENC) Yaounde Cameroon; ^4^ Department of Internal Medicine Cardiology Unit Yaounde Central Hospital Yaounde Cameroon; ^5^ Intensive Care Unit Yaounde University Teaching Hospital Yaounde Cameroon; ^6^ Yaounde Emergency Centre (CURY) Yaounde Cameroon

**Keywords:** Africa, biventricular noncompaction, cardiomyopathy, heart failure, hypertrabeculation

## Abstract

Ventricular noncompaction or hypertrabeculation is rare and unclassified cardiomyopathy that mostly affects the left ventricle. We report the case of biventricular hypertrabeculation in a 54‐year‐old woman who presented with congestive heart failure de novo associated with arrhythmia in a low‐income setting. We also discussed the therapeutic challenges.

## Graphical abstract

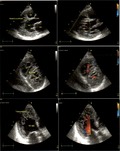

## 1 INTRODUCTION

Ventricular noncompaction or hypertrabeculations syndrome is rare nonclassified cardiomyopathy characterized by a thickened and spongy endocardium, and a thin and compact subendocardial layer. It mostly affects the left ventricle (LV), but the right ventricle (RV) disease is not exceptional. It could be congenital or acquired, isolated or associated with other structural congenital heart diseases as well as extra‐cardiac organs such as skeletal myopathy.^1^, ^2^ It could be asymptomatic or symptomatic, usually with a poor prognosis in symptomatic cases.^3^ There is a high genetic heterogeneity associated with the disease.^4^, ^5^, ^6^ The diagnosis can easily be made on cardiac ultrasound. Three main complications are associated with the disease—heart failure (often progressive), thromboembolic events, and arrhythmia with sudden death. We report the case of biventricular hypertrabeculations presenting with severe heart failure and arrhythmia in a sub‐Saharan African setting.

## 2 CASE PRESENTATION

Mme BH, a 54‐year‐old widow, was referred to our consultation by a radiologist for further evaluation of cardiomegaly and subpulmonary edema on chest X‐ray, and congestive liver on ultrasound. She complained of cough, abdominal pains, and fatigue of gradual onset for over 2 weeks. Her family history was remarkable for heart disease and sudden death in her late father. She is not a known diabetic patient. She denied drinking alcohol or use tobacco products. On clinical examination, she was short of breath with signs of central and peripheral fluid congestion. Vital parameters showed: BP: 155/82 mm Hg, resting HR: 87/min, RR: 16 cycles/min, SaO_2_: 99% on ambient air, weight: 80.7 Kg, height: 1.61 m, BMI: 31.1 kg/m^2^, and abdominal circumference: 105 cm. She had acanthosis nigricans around her neck. Our clinical diagnosis was de novo congestive heart failure. Complementary tests were performed.

### 2.1 Echocardiogram

**Figure 1:**
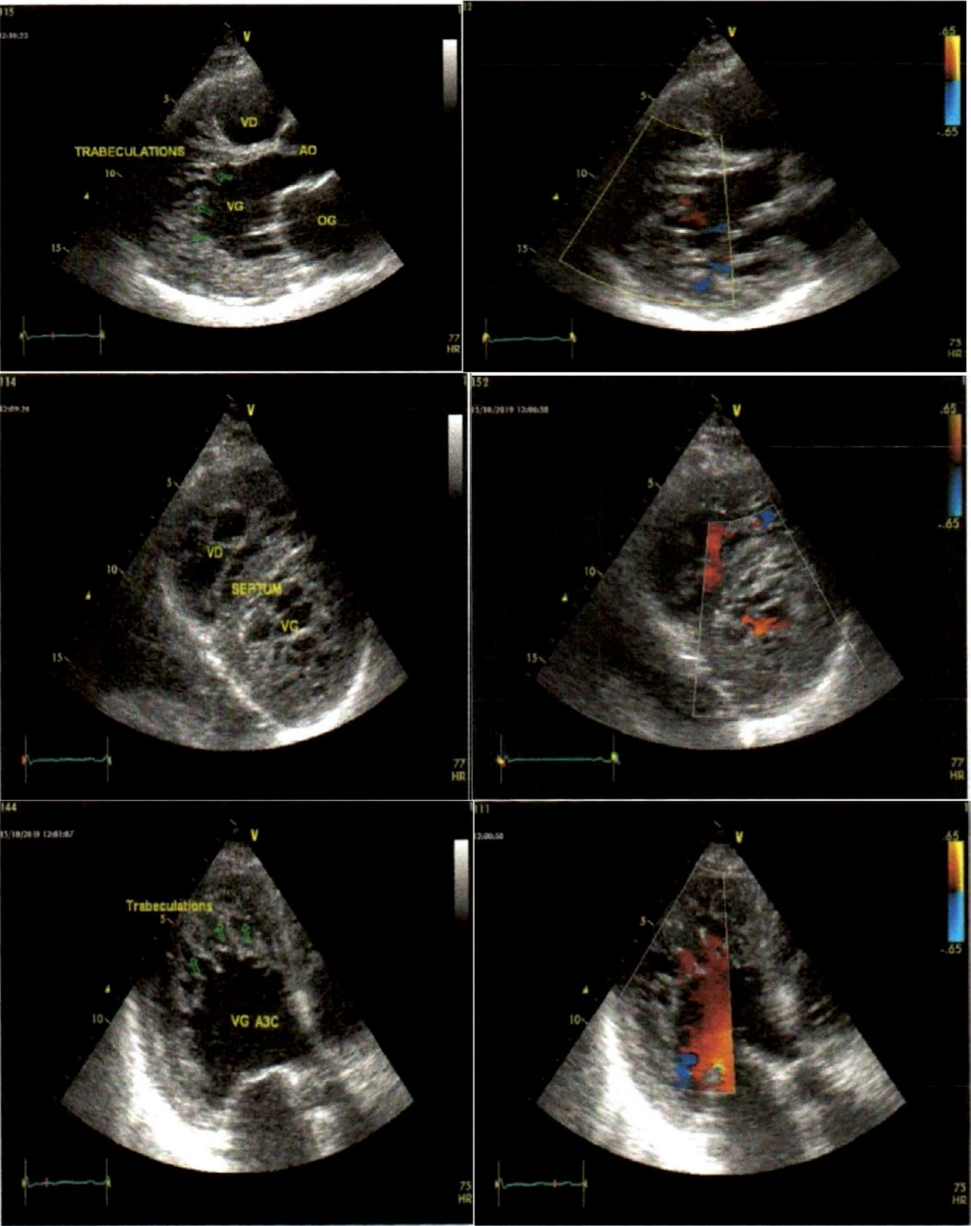
2D echocardiogram of the left ventricle. Upper panel shows the parasternal long axis with prominent trabeculations with Doppler within the recesses. The middle panel shows the parasternal short‐axis view of both ventricles with prominent trabeculations and intrarecess Doppler. The lower panel shows the apical 3‐chamber view of the left ventricle with prominent trabeculations and intratrabecular blood flow

**Figure 2:**
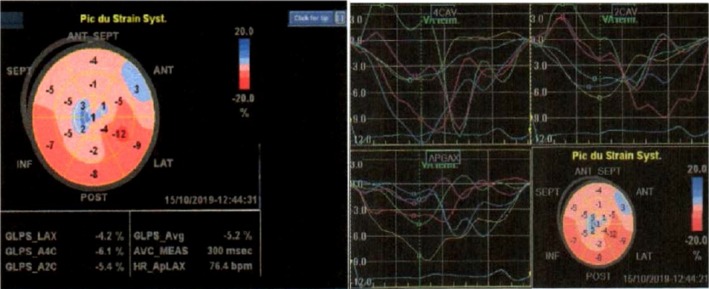
Global longitudinal LV strain showing a global and segmental dysfunction (left), with marked asynchrony (right)

**Figure 3:**
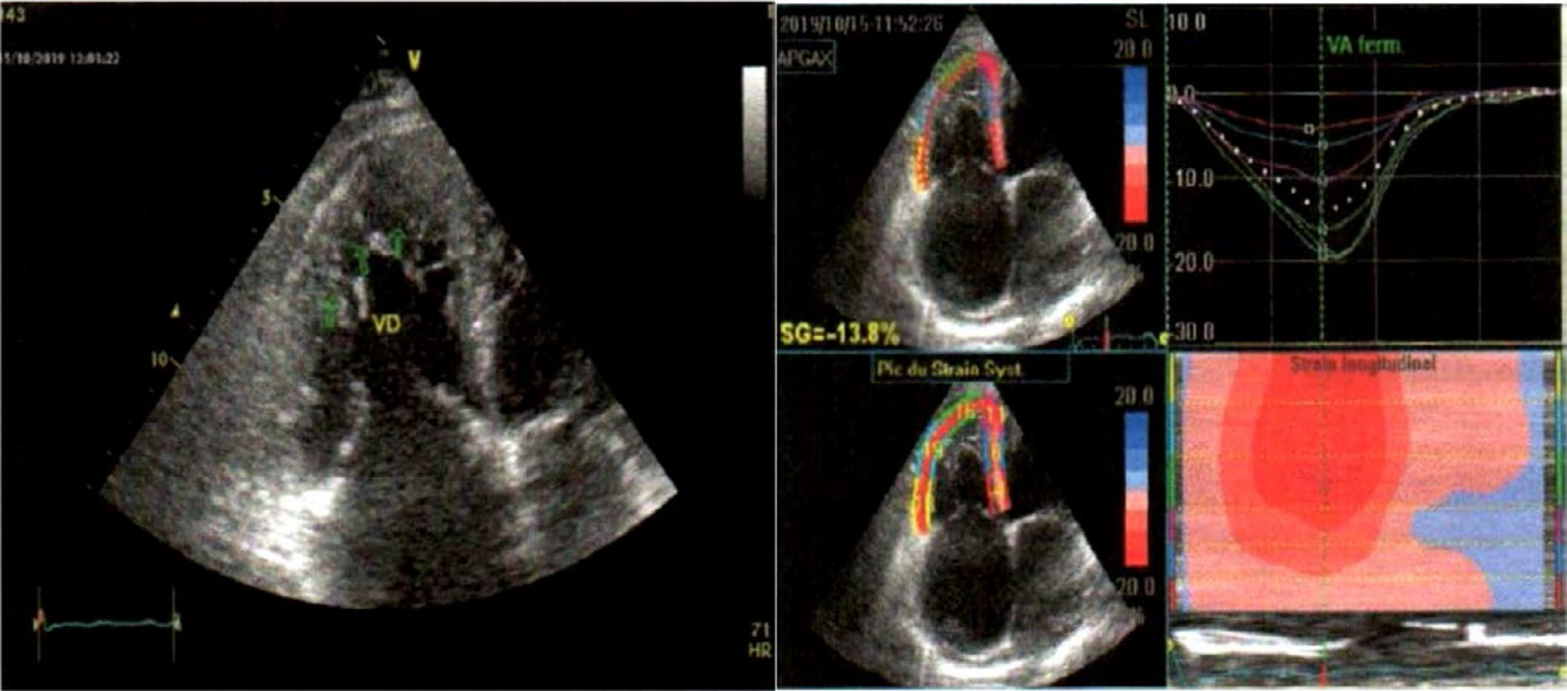
Hypertrabeculations of the right ventricle (left) with poor function (right)

There was a fusiform dilation of the ascending aorta (maximum diameter of 47 mm). The left ventricle (LV) was moderately dilated (end‐diastolic diameter: 38.3 mm/m^2^ and end‐diastolic volume index: 107 mL/m^2^) with prominent apical and lateral endocardial trabeculations with recesses, which communicated with the LV cavity on color Doppler studies (Figure 1). The subepicardial layer of the LV myocardium appeared normal. The ratio of the trabeculations to the subendocardial layer ranged from 3 to 5.3. The global LV global and segmental systolic functions were markedly reduced (LV ejection fraction by Simpson biplane: 30%, cardiac output: 3.37 L/min, cardiac index: 1.79 L/min, global longitudinal LV strain:‐5.2%), with marked segmental asynchrony (Figure 2). There was a restrictive mitral inflow pattern (E/A ratio: 2.43. E/e: 22.9. PCWP: 30 mm Hg). The left atrium (LA) was dilated (LA diameter: 48 mm. LA area: 27.2 cm^2^. LA volume: 47.4 mL/m^2^). The right ventricle (RV) was dilated with prominent apical endocardial trabeculations with recesses that communicated with the RV cavity (Figure 3). The subendocardial RV myocardium appeared normal. The global and regional RV systolic functions were reduced (TAPSE: 12 mm; RV area shortening: 33%; RV global strain: −13.8%; and RV free wall strain: −17%) (Figure 3). The right atrium (RA) was dilated (RA area: 31.6 cm^2^; RA volume index: 65.4 mL/m^2^). The pulmonary trunk was dilated (diameter: 36 mm). There was severe tricuspid regurgitation with laminar flow (RV‐RA maximum gradient: 30 mm Hg). There was moderate mitral valve regurgitation. There was mild to moderate aortic and pulmonary valve regurgitation. All the valves were thickened but pliable. The pulmonary pressure was moderately elevated and was associated with raised pulmonary vascular resistance (PVR) (estimated PAPs: 50 mm Hg; estimated PVR: 3.4 WU; and short pulmonary acceleration time with mid‐systolic notching). There was mild circumferential pericardial effusion. The inferior vena cava was markedly dilated (33 mm) and noncompliant (collapsing index <50%). Multiple ventricular ectopic beats were observed in the course of the cardiac ultrasound. Overall, the echocardiographic diagnosis was consistent with biventricular noncompaction or hypertrabeculations syndrome (VHT).

### 2.2 Electrocardiogram

**Figure 4:**
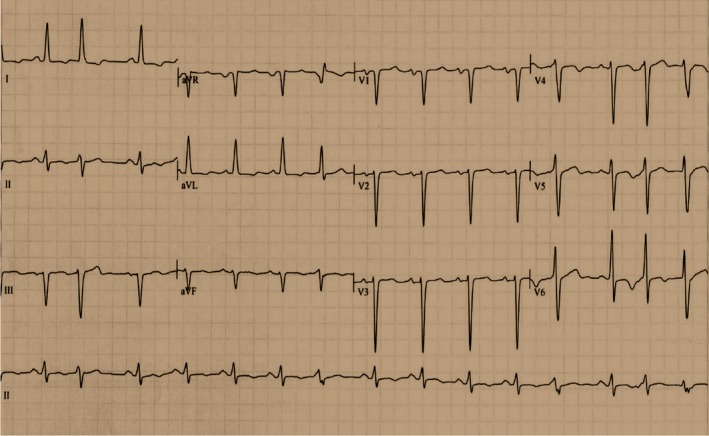
The ECG recording shows: sinus rhythm with frequent premature ventricular contractions and fusion beats. The ventricular response is 93 cycles/minute. Mild left axis deviation (−24°) with a large intraventricular gradient (217°). LV hypertrophy (Cornell: 34 mm) and LA dilation (P‐wave duration: 130 ms. Moritz sign +). Poor R‐wave progression. T waves were inverted in leads D1, D2, aVL, V3, and V4. Nonspecific intraventricular conduction delay. QTc is prolonged (QTc: 514 ms)

The recording (Figure 4) showed sinus rhythm with frequent premature ventricular contractions and fusion beats. The ventricular response was 93 cycles/min. There was a mild left axis deviation (−24°) with a large intraventricular gradient (217°). There was LV hypertrophy (Cornell index: 34 mm) and LA dilation (P‐wave duration: 130 ms. Moritz sign +). There was a poor R‐wave progression. T waves were inverted in leads D1, D2, aVL, V3, and V4. There was a nonspecific intraventricular conduction delay. The QTc was prolonged (QTc: 514 ms).

### 2.3 Biochemical profile

Blood tests showed Hb: 13.2 g/dL with microcytosis and hypochromia, normal platelets, and white cell counts; glycemia: 1.09 g/L; urea: 0.49 g/L; creatinine: 11.2 mg/L; uric acid: 82 mg/L; sodium: 148 mmol/L; potassium: 3.4 mmol/L; chloride: 105 mmol/L; calcium: 99 mg/L; magnesium: 22 mg/L; phosphorus: 39 mg/L; total cholesterol: 1.5 g/L; LDLc: 0.99 g/L; HDLc: 0.47 g/L; triglycerides: 0.74 g/L; ALAT: 163 IU/L; and ASAT: 94 IU/L. Urine was positive for albumin (0.81 g/L).

### 2.4 Treatment plan and evolution

She was put on standard treatment for heart failure (perindopril 5 mg daily; bisoprolol: 1.25 mg daily bedtime; aspirin 100 mg daily; furosemide 40 mg daily; spironolactone: 25 mg daily; and trimetazidine 35 mg BID). Subsequent follow‐up visits showed marked improvement in her symptoms and normalization of her blood pressure. She presented to our consultation four months after diagnosis with complaints: orthopnea, grade 2 shortness of breath, and mildly productive cough evolving for one week prior to the consultation. She had discontinued her diuretics two weeks earlier. Clinical evaluation showed signs of central and peripheral congestion. She had gained 3 kg of weight. We reintroduced and up‐titrated her diuretics, switched her aspirin to antivitamin K, and up‐titrated her bisoprolol to 2.5 mg daily. The rest of her medicines were unchanged.

## 3 DISCUSSION

Most cases of ventricular hypertrabeculation described in the literature have mostly involved the left ventricle.^1^, ^2^ We report the case of LV hypertrabeculation (LVHT) with classic echographic diagnostic characteristics. We also noticed intense RV hypertrabeculations with recesses fulfilling the echographic diagnostic criteria. This was associated with marked RV remodeling and dysfunction. Thus, our patient had biventricular hypertrabeculation syndrome. Biventricular involvement is seen in less than 50% of patients.^7^ An interesting point with this case that has not been described is the associated marked dilation of the great vessels of the heart. There was fusiform dilation of the ascending aorta and the pulmonary trunk. It is not clear whether this is a continuum of the disease or could be associated with pressure overload. Our patient had grade 1 hypertension at diagnosis that rapidly normalized with small doses of heart failure therapy. However, the relation between the degree of pressure overload and aortic size is not well established. The pulmonary trunk was also markedly dilated. The pulmonary systolic pressure was moderately elevated (underestimate using the TR jet velocity) with raised pulmonary vascular resistance. This could contribute to the dilation of the pulmonary trunk. Histologic features of the great vessels in patients with ventricular noncompaction syndrome should be studied for the presence of noncompaction in the tunica muscularis‐sarcomere involvement.^6^ In this case, we have described the strain pattern of the compact myocardium in both ventricles. This was markedly altered with marked segmental asynchrony.

Treatment in this patient was challenging. We offered her standard treatment of heart failure and noticed noncompliance with diuretic pills. The patient needs oral anticoagulation therapy due to the high thromboembolic risk. NOACs are expensive for this patient (widow). There is a concern with compliance in regular INR checks on antivitamin K. This patient also fulfilled criteria for implantable cardioverter‐defibrillation (ICD) device placement to prevent sudden death from fatal arrhythmia. This is not available and not accessible in our setting.^1^, ^2^, ^8^ There was marked segmental asynchrony on strain study but the QRS duration was <120 ms, and there was no LBBB. It is not known whether resynchronization could improve outcomes in such cases based on strain criteria. The patient also presented with the metabolic syndrome trait which will also require management. The prognosis in this patient is poor as we cannot halt progression of her heart failure.^3^

A limitation of this report is the lack of genetic studies which is not available and affordable in our setting. Relatives of the patient were not available for screening.

## CONFLICT OF INTEREST

None to declare.

## AUTHOR CONTRIBUTIONS

AMJ, LMK, HH, MP, and WYE: involved in echo diagnosis and patient care. AMJ, LMK, HH, SNA, BJ, and BH: drafted of the manuscript.

## ETHICAL APPROVAL

The patient gave her approval for her case to be published.
